# Impact of glass shape on time taken to drink a soft drink: A laboratory-based experiment

**DOI:** 10.1371/journal.pone.0202793

**Published:** 2018-08-27

**Authors:** Tess Langfield, Rachel Pechey, Mark Pilling, Theresa M. Marteau

**Affiliations:** Behaviour and Health Research Unit, Institute of Public Health, University of Cambridge, Cambridge, United Kingdom; Federation University Australia, AUSTRALIA

## Abstract

**Background:**

Glassware design may affect drinking behaviour for alcoholic beverages, with glass shape and size influencing drinking speed and amount consumed. Uncertainty remains both about the extent to which these effects are restricted to alcohol and the underlying mechanisms. The primary aim of the current study was to examine the effect of differently shaped glasses on time taken to drink a soft drink. The secondary aim was to develop hypotheses about mechanisms concerning micro-drinking behaviours and perceptual effects.

**Method:**

In a single-session experiment, 162 participants were randomised to receive 330ml of carbonated apple juice in a glass that was either inward-sloped, straight-sided, or outward-sloped. The primary outcome measure was total drinking time. Secondary outcome measures included micro-drinking behaviours (sip size, sip duration, interval duration), and perceptual measures (midpoint bias, drink enjoyment).

**Results:**

Participants drank 21.4% faster from the outward-sloped glass than from the straight-sided glass [95%CI: 0.2%,38.0%] in adjusted models. They were also 18.2% faster from the inward-sloped glass than the straight-sided glass, but this did not reach statistical significance with wide confidence intervals also consistent with slower drinking [95%CI: -3.8%,35.6%]. Larger sips were associated with faster drinking times (Pearson’s *r*(162) = -.45, *p* < .001). The direction of effects suggested sips were larger from the outward-sloped and inward-sloped glasses, compared to the straight-sided glass (15.1%, 95%CI: -4.3%,38.0%; 19.4%, 95%CI: -0.5%,43.6%, respectively). There were no significant differences between glasses in mean sip or interval duration. Bias in midpoint estimation was greater for the outward-sloped glass (12.9ml, 95%CI: 6.6ml,19.2ml) than for the straight-sided glass, although the degree of bias was not associated with total drinking time (Pearson’s *r*(162) = 0.01, *p* = .87).

**Discussion:**

Individuals drank a soft drink more quickly from an outward-sloped glass, relative to a straight-sided glass. Micro-drinking behaviours, such as sip size, are promising candidates for underlying mechanisms.

## Introduction

Overconsumption of sugary drinks and alcohol is a major public health concern, contributing to rising levels of obesity and premature preventable mortality and ill health [1–3]. Interventions that inform people of the health risks associated with their behaviour are generally ineffective at changing their behaviour [4]. When delivered as part of more intensive behaviour change programmes, they lack the reach required to change health at a population level, potentially widening health inequalities through drawing on cognitive resources that tend to be more readily available in those who are less rather than more deprived [5,6]. There is thus increasing policy interest in ‘choice architecture’ interventions [7], which, through changing cues in the environments in which choices are made, are hypothesised to change behaviour without drawing upon our limited cognitive resources [8]. These interventions are thought to work through automatic processes, without relying much on conscious engagement or individual agency [4,5].

Popularised in the book ‘Nudge’, some examples of such interventions include introducing chevrons on roads to reduce driving speed, adding flies on urinals to improve aim, and increasing the time taken for lift doors to close to encourage stair use [8]. To change health behaviours, reducing consumption or selection of less healthy foods and drinks might involve reducing tableware size [9], altering the proximity of healthier snacks in a cafeteria environment [10], reducing the availability of unhealthier snacks in a vending machine [11], and so on. For recent efforts to characterise these and other ‘choice architecture’ interventions see the TIPPME categorisation [12,13].

When we drink, we nearly always come into contact with a drinks container. Glassware, a modifiable cue in our drinking environment, is therefore a good candidate for interventions that aim to change drinking behaviour at a population level. It is widely documented that the design of a drinks vessel can influence subjective ratings of its contents, including flavour, perceived volume, liking for the drink, the amount an individual is willing to pay (for recent reviews see [14,15]), and taste expectations [16]. The extent to which these perceptual effects influence drinking behaviour is perhaps less certain. However, with growing evidence that the design of glass and tableware can influence the amount consumed, for food and non-alcoholic drinks [17], as well as for wine [18,19], understanding the mechanisms behind these effects is key to optimising interventions to reduce consumption.

There is some evidence that glass shape influences drinking speed. Attwood and colleagues [20] found that individuals consumed full portions of beer 60% slower from straight, compared with curved, beer glasses, although no differences were found for smaller portions, or for a soft drink. These authors attributed the findings to biased midpoint estimation, which, their results indicated, was greater for curved glasses, attributed to the nonlinear relationship between height and volume. However, there was only a trend towards an association between the degree of this perceptual bias and rate of consumption, suggesting that other mechanisms may have contributed to the differences in drinking rate. Indeed, a subsequent study from the same group investigated whether labelling the glass with volume markers (at ¼, ½, ¾) could slow consumption rate from curved glasses [21]. Findings suggested slower drinking from clearly-labelled glasses, relative to unmarked glasses, but the confidence intervals were wide and also consistent with faster drinking. Further research is warranted to explore the extent to which the effect found in this initial study is limited to alcoholic-drinks, as well as to further understand the role of biased volume estimation in influencing drinking behaviour.

An additional or alternative mechanism that may contribute to the effects of glass shape on drinking rate could be the cueing or affordance of ‘micro-drinking behaviours’. These reflect the micro-structure of drinking behaviour, and include sip size (volume consumed in each sip), sip durations (length of time spent per sip), interval durations (length of time in-between sips), and drinking ‘tempo’ (the dynamic pattern of drinking rate across the drinking episode). Two studies report people taking larger sips from larger cups [22,23] although confounding of cup size with other variables in these studies mean it was not clear whether differences were due to effects of the container size (as interpreted by Lawless and colleagues [22]), portion size (known to impact *ad libitum* consumption; for a review see [24]), or drinking context, namely, whether drinking was ‘instructed’ or ‘natural’ (as interpreted by Bennett and colleagues [23]). Two further studies also reported a greater number of sips and slower drinking from larger vs smaller wine glasses [25] and straight vs curved beer glasses [20].

Glasses might ‘afford’ sipping behaviours based on physical properties of the glass and the liquid it contains. Glasses with a curved, or outward-sloped design (as used in [20] and [21]) may automatically cue larger sips, relative to straight-sided glasses, due to the increased flow of liquid across a wider circumference when glasses are tilted to the same degree. Related to this, individuals might take large initial sips from outward sloped glasses to avoid spillages. There may be an additional effect of the volume of liquid contained in the glass, or glass fullness. For glasses that are relatively empty, a larger angle of tilt is generally required to drain the drink, but for full glasses, relatively less effort is required. Exploring how micro-drinking behaviours change over time might therefore be informative. A recent study by Cliceri and colleagues [26] identified dynamic drinking patterns (decelerated or accelerated), and compared these between two glass types (tall/narrow vs short/wide). Though both glasses led to a decelerated pattern, this pattern was more marked for the short/wide glass, characterised by a greater proportion consumed in the first half of the drinking episode, as compared to the second half.

Taken together, the evidence suggests that micro-drinking behaviours might be important for understanding the effects of glass shape on drinking behaviour, but it is not clear which particular aspects of glass shape or size might cue differences in micro-drinking behaviours (for example, glass wall slope), nor which micro-drinking behaviours warrant further investigation (for example, sip size).

The present study investigated whether glass shape predicts total drinking time for a soft drink, using inward-sloped, straight-sided, and outward-sloped glasses. Although previous research was inconclusive as to the effect of glass shape on drinking time for a soft drink, with an effect found only for an alcoholic drink [20], the soft drink used was not matched visually to the alcoholic one (clear vs amber liquid). As such, we predicted that when using an amber liquid (matching the alcoholic drink used by Attwood and colleagues [20]), outward-sloped glasses would lead to faster consumption than straight-sided glasses, as Attwood et al. [20] found for the alcoholic drink. We also predicted that, conversely, inward-sloped glasses would lead to slower consumption than straight-sided glasses. We also explored micro-drinking behaviours (sip size, sip and interval duration) and perceptual effects (bias in midpoint estimation and drink enjoyment) as two possible underlying sets of mechanisms.

## Methods

### Participants

Participants were recruited from the students and staff at the University of Cambridge, as well as the general population, using flyers, mailing lists, and word of mouth (forming a convenience sample). To take part, it was required that individuals: were over 18 years old; had English as a first language or an equivalent level of fluency; were prepared to consume a drink that contained sugar (and to confirm that this would not cause them any difficulties with their health); and had no known allergies to Appletiser® (sparkling apple juice, with 33.7g sugar per serving). We calculated that in order to detect a medium overall effect size (*f* = .25) of glass shape on total drinking time, with 80% power, at an alpha level of .05, a sample of 159 was needed. To allow for equal numbers of males and females, a total sample size of *N* = 162 was sought.

### Study design

In a between-subjects design, participants were randomised to receive 330ml of Appletiser® served in one of three glasses: a) inward-sloped, b) straight-sided, or c) outward-sloped. Randomisation was constrained to ensure equal group sizes (*n* = 54), as well as equal proportions of males and females in each group (providing a more representative sample, and given the possible link between gender and drinking behaviours (e.g. [20,22,26]). The primary outcome measure was total drinking time (min), and secondary outcome measures included micro-drinking behaviours (mean sip size, mean sip duration, mean interval duration), and perceptual measures (bias in estimating midpoint of drink and drink enjoyment). The full study protocol was pre-registered and is available at https://osf.io/sj5wx/.

### Materials and measures

#### Glasses

**Fig 1:**
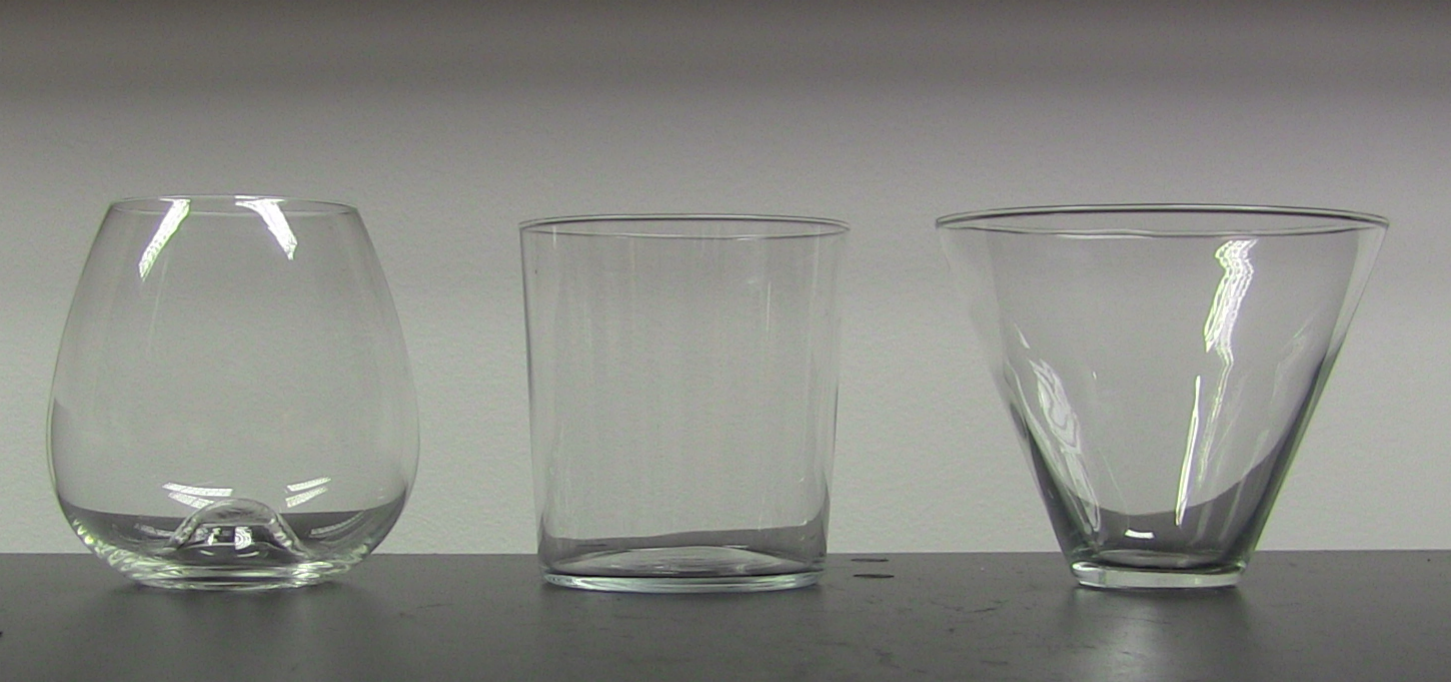
Glasses used in the study. From left to right, inward-sloped, straight-sided, outward-sloped.

The inward-sloped glass was designed by Dartington and supplied by www.havens.co.uk (height: 90mm, weight: 125g, capacity: 440ml, rim diameter: 62mm). The straight-sided glass was designed and supplied by LSA International https://www.lsa-international.com/ (height: 85mm, weight: 110g, capacity: 400ml, rim diameter: 85mm). The outward-sloped glass was designed by Libbey and supplied by www.drinkstuff.com (height: 89mm, weight: 170g, capacity: 400ml, rim diameter: 115mm). See Fig 1 for images of the glasses.

#### Primary outcome measure: Total drinking time

Total drinking time (min) was measured using video recordings of the drinking sessions. These video recordings were coded using a program to extract the time at each sip initiation and sip end (indicated by key press). Total drinking time was calculated by taking the difference in time between: (1) the initiation of the first sip (when the glass touches the lips for the first time), and (2) the endpoint of the last sip (when the glass leaves the lips for the final time).

#### Secondary outcome measures

Micro-drinking behaviours were measured from video recordings of the drinking sessions, coded manually using a program with key presses signalling the initiation and endpoint of sips.

*Mean sip size (ml)*, or the average volume consumed per sip, was measured by dividing 330ml (total volume consumed in the task) by the number of sips (extracted from the coded video data).

*Mean sip duration (sec)*, or the average time spent sipping, was calculated by taking the average of all sip durations (the difference between the initiation and end point of a given sip), again derived from the coded video data.

*Mean interval duration (sec)*, or the average time spent in between sips, was calculated by taking the average of all inter-sip intervals (the difference between the endpoint of a given sip and initiation of the next sip), again derived from the coded video data.

*Bias in midpoint estimation* was determined from six poured estimates of the midpoint of the drink (165ml),averaged to provide a single estimate for each participant. This was then subtracted from 165ml (the true midpoint), to determine bias. Negative values reflect underestimation of the true midpoint (pouring too little liquid into the glass), while positive values indicate overestimation of the true midpoint (pouring too much liquid into the glass).

*Drink enjoyment* was measured using two questions, rated on a ten-point scale anchored at one end by 1 (Not at all) and at the other end by 10 (Extremely), which asked how ‘pleasant’ and ‘tasty’ the drink was. Together, these ratings formed ‘drink enjoyment’ (Cronbach’s a = .90). These two questions formed part of a ‘taste perception’ task in which participants rated the drink along 10 different descriptors (‘fruity’, ‘smooth’, ‘sweet’, ‘refreshing’, ‘bitter’, ‘strong tasting’, ‘gassy’, ‘pleasant’, ‘light’ and ‘tasty’). The results of all other ratings were not analysed. This rating task and measure of ‘drink enjoyment’ has been used previously in a laboratory study investigating drinking behaviour [27].

#### Filler task

Participants completed a computer-based word-search task. They were asked to find as many words as possible in 4-minutes. This was included to obscure the true aim of the study, and to make the cover story (that we were investigating the impact of glucose on cognitive performance) more believable. The data from this filler task were not analysed.

#### Awareness of the purpose of the study

Participants were asked to indicate what they thought the main purpose of the study was, choosing from nine possible answers. Those who correctly identified ‘To investigate the impact of glass design on drinking rate’ were coded as aware.

### Procedure

The study was approved by the University of Cambridge Psychology Research Ethics Committee (reference: PRE.2017.018). Eligible participants were invited to attend a single study session, scheduled between 8 am and 8 pm, Monday to Saturday. As a cover story, participants were informed they were taking part in a study on the impact of glucose on cognitive performance. On arrival, participants completed self-reported eligibility screening, and gave written informed consent. They then answered questions about their age, gender, level of education, and thirst. During this time, the experimenter removed a 330ml can of Appletiser® from the fridge, and brought it into the testing room along with the glass the participant had been randomised to drink from. The full 330ml can was then poured into the glass, immediately prior to serving to ensure consistent carbonation across study sessions. The experimenter then placed the drink on a coaster and informed the participant to consume the drink at their own pace, whilst watching a nature documentary (“River without Frontiers: The Secrets of Nature”, 2008). Before leaving the room, the experimenter turned on the documentary and switched on the video camera. Participants were asked to open the door when they had finished the drink.

Next, participants completed the computer-based four minute word-search, followed by the ‘taste perception’ task, in which they rated the drink along 10 descriptors. For the final task, assessing bias in midpoint estimation, the experimenter first placed the 330ml glass of Appletiser® directly in front of the participant—the same glass as they had been randomised to drink from. Participants were then instructed to pour half of the liquid in the glass into a jug containing 660ml of Appletiser®, which was placed behind the glass. After the experimenter had weighed the glass to determine the participant’s poured estimate, the participant was instructed to pour another midpoint estimate, this time from the jug into an empty glass. Pours were attempted six times in total (three from the glass into the jug, and three from the jug into the glass). For reference, they were presented with a full 330ml glass of Appletiser® (a ‘Reference Glass’), placed to their left throughout the task.

Finally, participants were asked to indicate what they thought the aim of the study was, to examine the effectiveness of the cover story in blinding participants to the behavioural measures and the true nature of the study. A basic debrief was provided, and a full debrief was sent via email once testing for the study was complete. Participants received £7 in cash for taking part.

### Statistical analysis

The primary analysis was a multiple linear regression to determine whether glass shape (inward-sloped, straight-sided, outward-sloped) predicted drinking rate. Using linear regression, two dummy-variables (inward-sloped, outward-sloped) were entered, with straight-sided as the reference variable. Analyses were run to adjust for differences in gender, age, thirst, maximum daily temperature, and time of day. Secondary analyses were conducted to determine whether glass shape predicted micro-drinking behaviours (mean sip size, mean sip duration, mean interval duration), as well as perceptual factors (bias in midpoint estimation, drink enjoyment). Sensitivity analyses excluded participants who correctly guessed the true purpose of the study. Reliability analyses, with an independent coder, were conducted on 20% of the videos.

The data for this experiment are available from the Open Science Framework: https://osf.io/fwmg9/ (DOI: 10.17605/OSF.IO/FWMG9).

## Results

### Baseline characteristics

One hundred and sixty two individuals took part in the study (50% female). The mean age was 24.0 years (*SD* = 6.72, range = 18 to 69), and all participants had at least AS/A Level education (40.7% had AS/A Level, 34.6% had an undergraduate degree, and 24.7% had a postgraduate degree). Baseline characteristics, split by condition, are given in Table 1.

**Table 1:** Baseline characteristics of participants and covariates, by group.

	Inward-sloped (*n* = 54)	Straight-sided (*n* = 54)	Outward-sloped (*n* = 54)	Total sample (*N* = 162)
Female (*n*)	27	27	27	81
Age	23.0 (5.05)	24.2 (7.14)	24.7 (7.70)	24.0 (6.72)
Thirst (1–10)	6.09 (1.40)	5.83 (1.69)	6.20 (1.48)	6.04 (1.53)
Maximum daily temperature (^o^ C)	19.6 (3.64)	20.4 (3.90)	20.2 (4.00)	20.1 (3.84)
Time of day (hours after midday)	1.43 (3.17)	1.50 (2.54)	1.26 (2.89)	1.40 (2.86)

*Note*. Values given are mean (*SD*).

### Primary analysis

#### Impact of glass shape on total drinking time

Visual inspection of the distributions indicated positive skew in total drinking time. The primary analyses were therefore conducted on log10 transformed (total drinking time). Where means are reported, they are geometric (back transformed) with 95% CIs [28,29]. We note that the actual effect size for an overall ANOVA was *f* = .20, *p* = .08, which is smaller than the assumed effect size in the sample size statement. The equivalent regression analysis was preferred, to explicitly estimate the pairwise contrasts. Regression model diagnostics were checked and were acceptable.

**Fig 2:**
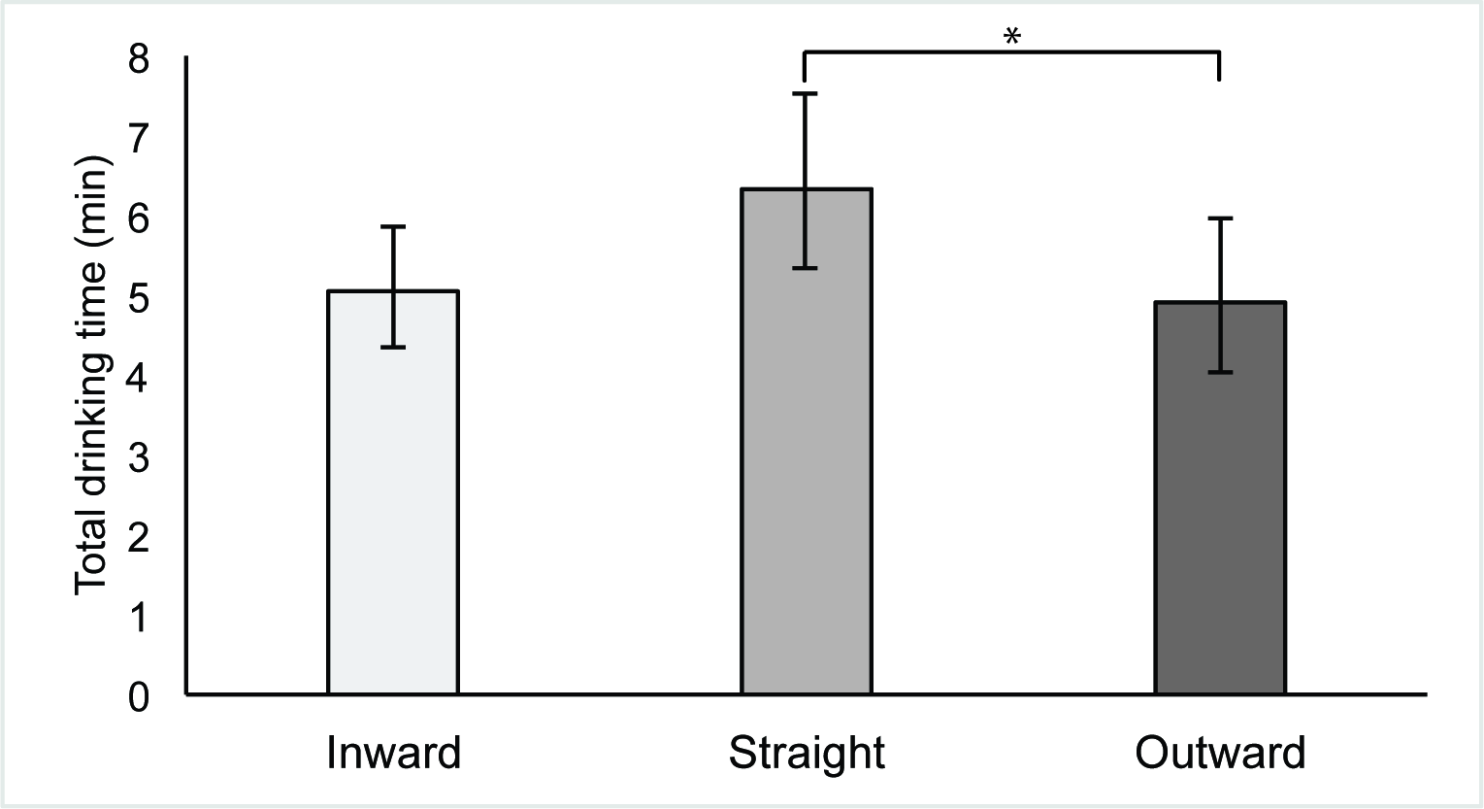
Drinking time (unadjusted geometric mean) and glass shape. {Error bars show back transformed 95% CIs. * reflects significance at *p* < .05 level}.

Individuals drank slowest from the straight-sided glass (*Mdn* = 5 minutes 48 seconds, *IQR* = 5 minutes 53 seconds), fastest from the outward-sloped glass (*Mdn* = 4 minutes 46 seconds, *IQR* = 5 minutes 34 seconds) and between these two speeds from the inward-sloped glass (*Mdn* = 5 minutes 26 seconds, *IQR* = 3 minutes 38 seconds). See Fig 2 for geometric mean total drinking time by condition.

Males drank faster than females taking, on average, 4 minutes 35 seconds (95% CI: 3 minutes 56 seconds, 5 minutes 20 seconds) and 6 minutes 22 seconds (95% CI: 5 minutes 37 seconds, 7 minutes 12 seconds) respectively, *p* = .001.

Adjusting for gender and other pre-specified covariates, total drinking time from the outward-sloped glass was 21.36% faster than from the straight-sided glass, (95% *CI*: 0.21%, 38.02%), *p* = .048. Total drinking time from the inward-sloped glass was 18.22% faster than from the straight glass, although this was also consistent with slower drinking (95% *CI*: -3.81%, 35.57%), *p* = .098. See Table 2 for adjusted and unadjusted models.

**Table 2:** Unadjusted (univariate) and adjusted (multivariate) regression, predicting log10(total drinking time).

	Unadjusted regression analyses					Adjusted regression analyses			
Independent variable	*B*	Exp(*B*)	95% CI Exp(*B*)	*p*-value	R^2^	*B*	Exp(*B*)	95% CI Exp(*B*)	*p*-value
(Constant)	-	-	-	-	-	0.685	4.839	2.367 to 9.895	< .0001
**Glass shape**					.031				
Inward-sloped	-0.098	0.798	0.625 to 1.018	.069		-0.087	0.818	0.644 to 1.038	.098
Outward-sloped	-0.110	0.776	0.608 to 0.989	.041		-0.104	0.786	0.620 to 0.998	.048
**Gender**					.065				
Female	0.143	1.389	1.143 to 1.688	.001		0.145	1.397	1.145 to 1.706	.001
**Age**	0.0022	1.005	0.991 to 1.020	.51	.003	0.002	1.005	0.990 to 1.019	.52
**Thirst (1–10)**	-0.016	0.965	0.903 to 1.030	.28	.007	-0.018	0.959	0.899 to 1.023	.20
**Maximum daily temperature (**°**C)**	0.0090	1.021	0.995 to 1.048	.12	.015	0.0053	1.012	0.986 to 1.039	.35
**Time of day (hours after midday)**	-0.0003	0.999	0.965 to 1.035	.97	.000008	-0.0047	0.989	0.956 to 1.024	.53

*Note*. Adjusted analyses: *F*(7,154) = 2.85, *p* = .008, R^2^ = .115. Exp = Power of 10.

### Secondary analyses

#### Glass shape and micro-drinking behaviours

Visual inspection of the frequency distributions of micro-drinking behaviours (mean sip size, mean sip duration, mean interval duration) indicated positive skew. Log(10) transformations improved the shape of these distributions so analyses were conducted on the transformed data for all three micro-drinking behaviours. For micro-drinking behaviours, where means are reported, these are Geometric (back transformed) with 95% CIs. See Table 3 for medians (IQRs) of micro-drinking behaviours, as well as mean (SD) for perceptual measures, for each glass shape.

**Table 3:** Summary of secondary outcome measures, split by condition.

		Inward	Straight	Outward
**Micro-drinking behaviours**	Mean sip size (ml)^a^	22.00 (16.50 to 30.00)	18.33 (12.11 to 25.91)	19.41 (15.00 to 30.75)
	Mean sip duration (sec)^a^	2.14 (1.72 to 2.89)	1.94 (1.47 to 2.72)	1.93 (1.65 to 2.80)
	Mean interval duration (sec)^a^	17.71 (11.77 to 29.46)	19.15 (11.26 to 38.34)	15.35 (11.76 to 23.50)
**Perceptual measures**	Bias in midpoint estimate (ml)^b^^,^ ^c^	-2.27 (21.05)	-3.00 (11.42)	-15.92 (15.95)
	Drink enjoyment (1–10)^b^	7.02 (1.65)	7.00 (1.68)	7.18 (1.66)

Note.

a. Due to positive skew in all micro-drinking behaviours, these values given are *Mdn* (*IQR*).

b. Drink enjoyment and bias in midpoint estimation are *M*(*SD*).

c. 0ml reflects 0 bias in estimation, negative values reflect underestimation of true midpoint, positive values reflect overestimation of true midpoint.

Females took smaller mean sips (*Geomean* = 17.79ml, 95% CI: 16.07ml, 19.68ml) than males (*Geomean* = 22.85ml, 95% CI: 20.41, 25.58), *t*(161) = 3.29, *p* = .001). To adjust for these differences, we included gender in the model for sip size.

Glass shape and gender explained 8.7% of the variability in log(10) mean sip size, *F*(3,158) = 5.01, *p* = .002. After adjusting for the effect of gender (B_Female_ = -0.11, *p* = .001), estimates suggested that individuals took sips that were 19.40% larger from the inward-sloped glass, compared to the straight-sided glass, although the data were also consistent with smaller sips (95% CI: -0.46%, 43.55%), *p* = .057. Sips were estimated to be 15.08% larger from the outward-sloped glass than from the straight-sided glass, although the data were again also consistent with smaller sips (95% *CI*: -4.28%, 38.04%), *p* = .13.

Unlike mean sip size, gender did not predict mean sip duration or mean interval duration. It was therefore not included in these models. Glass shape did not meaningfully predict log(10) mean sip duration (*F*(2,159) = 0.91, *p* = .40), or log(10) mean interval duration (*F*(2,159) = 0.39, *p* = .68).

**Fig 3:**
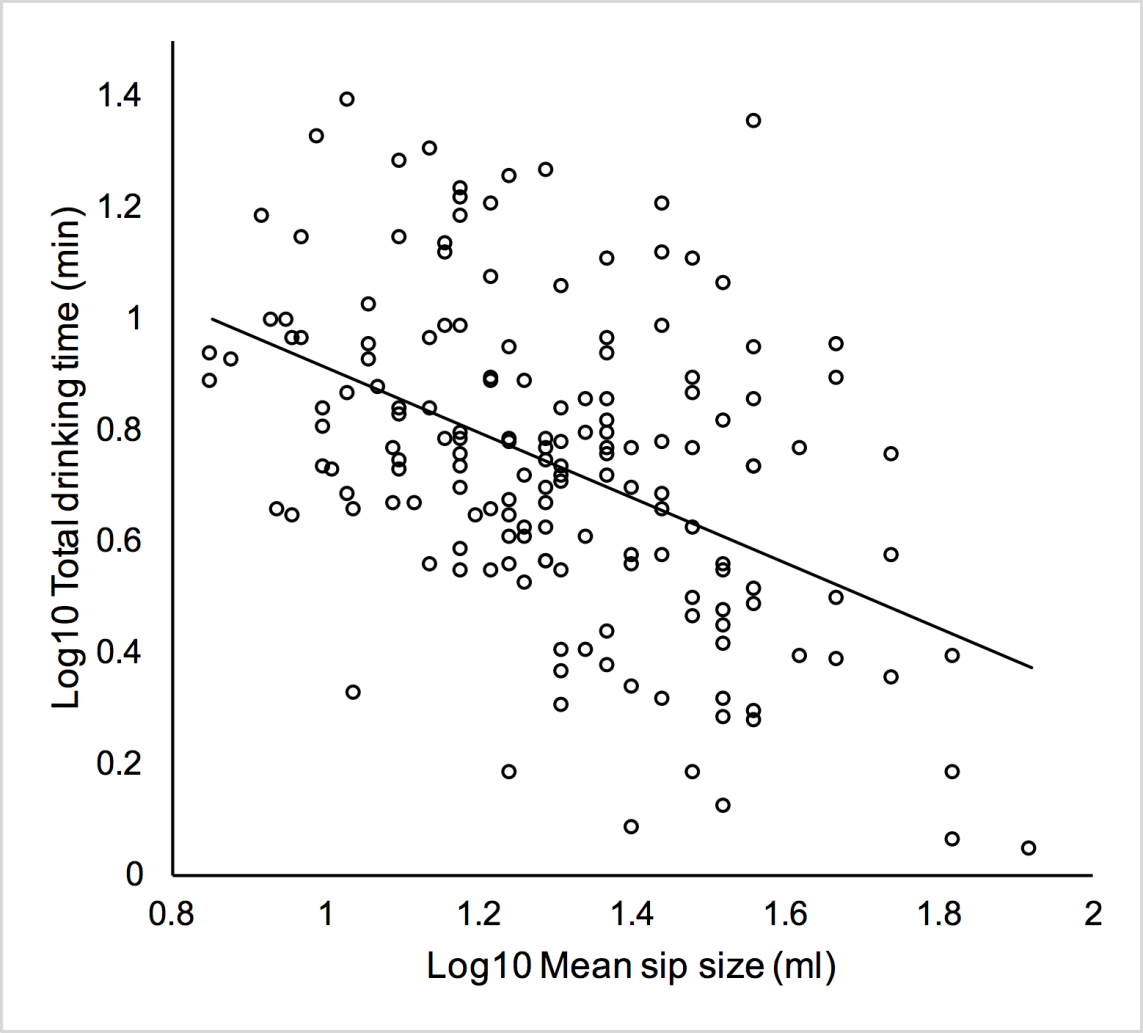
Relationship between log(10) transformed mean sip size and log(10) transformed total drinking time.

There was a medium-sized negative association between mean sip size and total drinking time, suggesting that larger mean sip sizes were associated with shorter total drinking times (Pearson’s *r*(162) = -.45, *p* < .001), see Fig 3. Mean sip duration was not associated with total drinking time (Pearson’s *r*(162) = -.09, *p* = .25).

#### Glass shape and drink midpoint estimation

**Fig 4:**
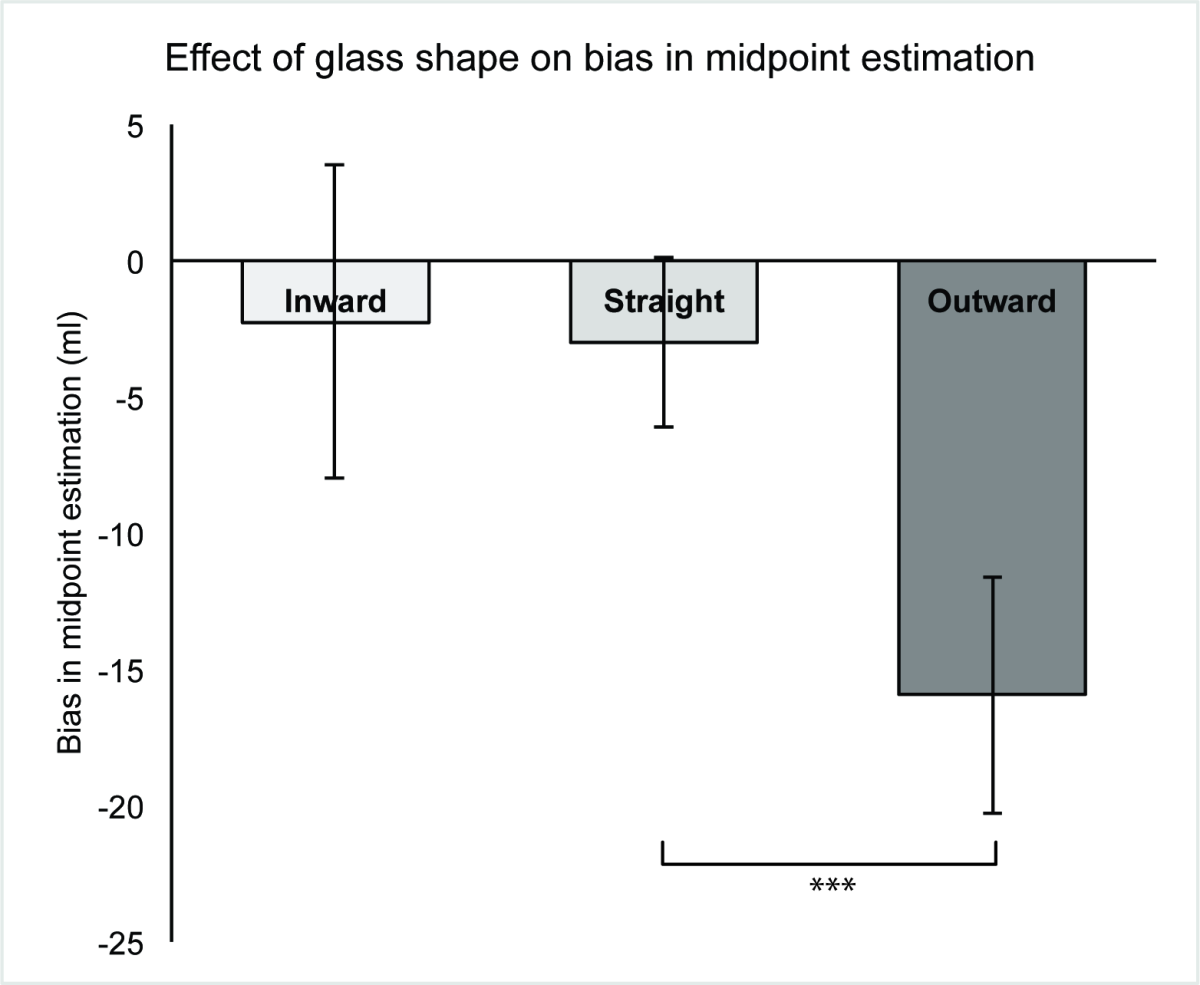
Mean bias in midpoint estimation and glass shape. **{**Error bars reflect 95% CIs. Negative numbers reflect under filling of glass when estimating midpoint. *** reflects significance at *p* < .001 level.}.

Glass shape predicted bias in midpoint estimation, explaining 12.7% of the variance, (*F*(2,159) = 11.54 , *p* < .001). All glasses were associated with an underestimation of the true midpoint (see Fig 4). Individuals poured 12.92ml less into an outward-sloped glass than into a straight-sided glass (95% CI: 6.61ml, 19.24ml), *p* < .001, suggesting greater underestimation of the midpoint, in line with using height as a cue for volume. Individuals poured 0.74ml more into an inward-sloped glass than into a straight-sided glass, but there was no evidence that this difference was meaningful (95% CI: -7.05ml, 5.58ml), *p* = .82. Bias in midpoint estimation was not associated with total drinking time (Pearson’s *r*(162) = 0.01, *p* = .87).

#### Glass shape and drink enjoyment

We found no evidence that glass shape influenced how much the drink was enjoyed (*F*(2,159) = 0.18, *p* = .83); means and standard deviations are given in Table 3. Drink enjoyment was not associated with log(10) total drinking time (Pearson’s *r*(162) = -.04, *p* = .62).

### Sensitivity analysis

Removing participants (*n* = 5) who correctly identified the purpose of the study (to investigate the impact of glass shape on drinking rate) did not alter the main conclusions. Unadjusted and adjusted estimates suggested outward-sloped glasses led to faster drinking than straight glasses (21.11%, 95% CI: -1.16%, 38.48%, *p* = .06; and 20.93%, 95% CI: -1.16%, 38.06%), *p* = .06, respectively), although the confidence interval for the outward-sloped vs. straight-sided glass comparison now crossed zero.

### Reliability analysis

Inter-rater reliability was high for the video coded data. Single measures intra-class correlation indicated strong and positive associations for total drinking time (32) = .98, *p* < .001, sip size (32) = 1.0, *p* < .001, sip duration (32) = .99, *p* < .001, and interval duration (32) = .99, *p* < .001.

### Exploratory analyses

**Fig 5:**
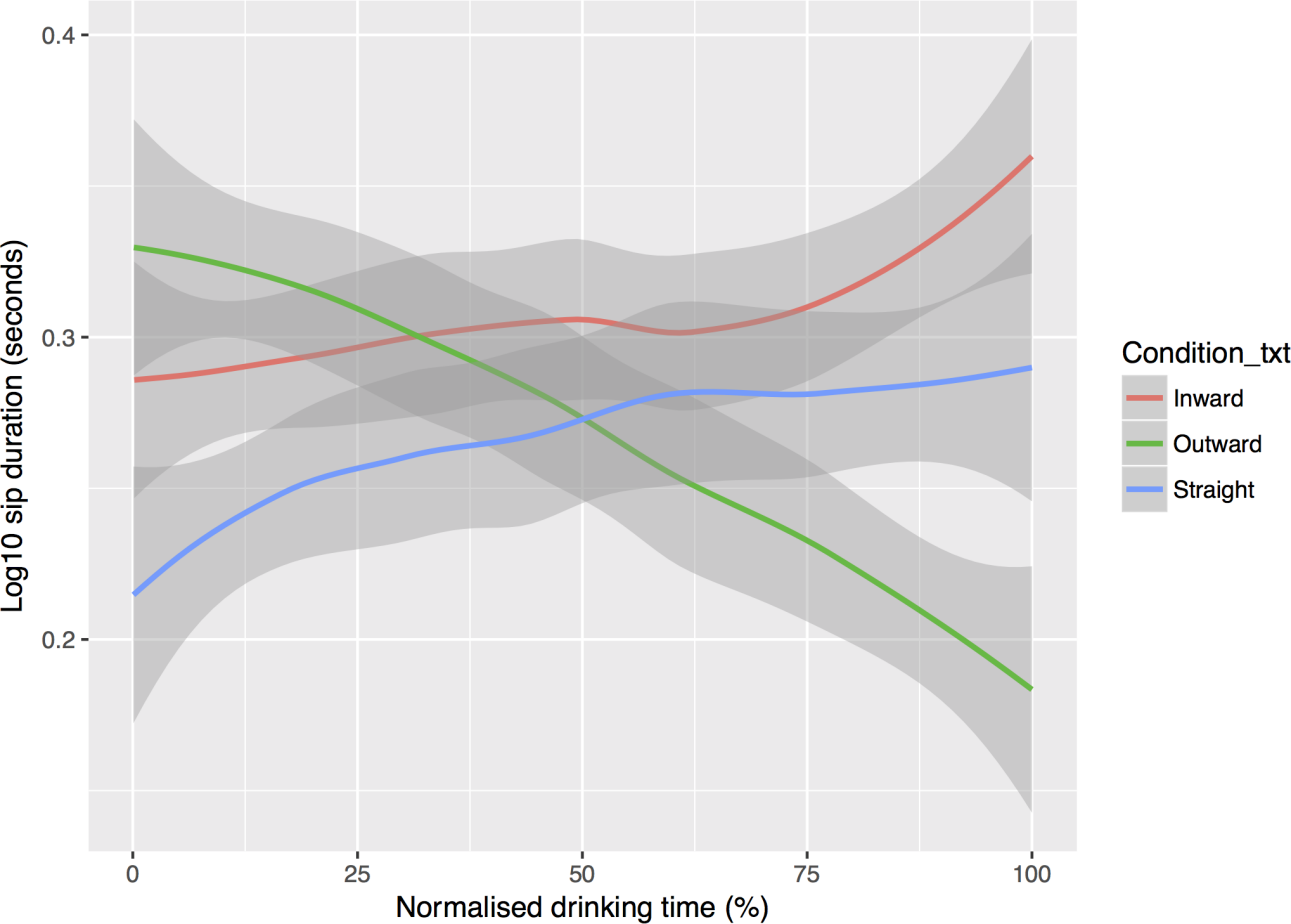
Change in sip durations across the drinking period, with LOESS smoothed lines. Total drinking time is normalised (%) to allow for between subject comparisons. Line is fitted with locally weighted scatterplot smoothing (LOESS) and the grey shading is the 95% confidence region.

To understand the dynamic pattern of drinking rate across time, or ‘drinking tempo’, we explored the coded video data further. We plotted individual log(10) sip durations as a function of time, separated by condition. Time was normalised to each participant’s total drinking time, with 100% reflecting each individual’s total drinking time (see Fig 5). Visual inspection of these data suggested that glass shape influenced drinking tempo. For the outward-sloped glasses, sip durations were longer at the start, and shorter towards the end, while for the straight-sided and inward-sloped glasses, sip durations slightly increased over time. A linear mixed effects regression of log(10) sip duration with individual as a random factor and a fixed interaction term showed that first sip durations were longer from the outward-sloped glass (*Geomean* = 2.47 seconds, 95% CI: 2.12, 2.88) than the straight glass (*Geomean* = 1.83 seconds, 95% CI: 1.53, 2.20), *p* = .004. First sip durations from the inward-sloped glass (*Geomean* = 2.13 seconds, 95% CI: 1.84, 2.47) did not differ from the straight-sided glass, *p* = .386. The pattern of sip durations over time (line gradient) also differed significantly between the outward-sloped glass and straight-sided glass (*p* < .0001). Note: means and 95% CIs are back-transformed from log10 scale and p-values are Kenward-Roger adjusted.

## Discussion

The present study found an effect of glass shape on drinking rate for a soft drink: faster drinking was observed from outward-sloped glasses than from straight-sided glasses. Findings for inward-sloped glasses were inconclusive, although suggestive of faster drinking when compared with straight-sided glasses. To our knowledge, this study is the first to show an effect of glass shape on total drinking time for a non-alcoholic drink, with a previous study suggesting the effect might have been limited to alcohol [20]. The findings are in line with a growing evidence base suggesting that altering cues in the environment (including glassware, tableware, and packaging) can influence associated behaviours (speed of consumption and amount consumed) [17–21], and thus provide evidence for the effectiveness of these interventions in ‘proximal physical micro-environments’, also known as ‘choice architecture’ interventions or nudging [12,13].

The micro-structure of drinking behaviour—micro-drinking behaviours—was considered as a possible mechanism underlying the effect. Mean sip size was negatively associated with total drinking time, with larger sips associated with faster consumption. Individuals tended to take larger sips when drinking from the inward-sloped and outward-sloped glass, as compared to the straight-sided glass. However, the confidence intervals for these effects crossed zero. Thus, it remains unclear whether differences in sip size taken from smaller vs larger cups shown in previous studies [22,23] are seen when drinking from glasses of different shapes. However, it is possible that ‘*mean* sip size’, which is derived from dividing the amount consumed by the total number of sips, may not capture the complexity of sipping behaviours, and miss true differences where they exist. These might be better examined dynamically, or across the drinking period. Given that we did not measure individual sip sizes, to examine the dynamic changes in drinking rate, we explored individual-level (rather than average) sip durations, and plotted these as a function of time. From the outward-sloped glass, participants took longer initial sips, which then got shorter over time. By contrast, from the straight-sided and inward-sloped glasses, sip durations gradually increased over time. Using sip duration as a proxy for sip size, we speculate that sips were initially larger from the outward-sloped glass due to an automatic cueing of sip size. When full, and given the same angle of tilt, an outward-sloped glass affords a faster flow of liquid than the other glasses. These larger initial sips might therefore be a key determinant in the faster overall drinking rate seen from outward-sloped glasses. Taken together, these findings suggest that drinking glasses may cue specific patterns of micro-drinking behaviours, depending on both glass shape and relative fullness which in turn may influence total drinking time.

In addition to micro-drinking behaviours, we explored perceptions as another set of possible mechanisms underlying the observed effect. While the ability to estimate volume was poorest in the outward-sloped glass, as previously shown [20,30], midpoint bias was unrelated to total drinking time in our study. One possibility is that individuals do not titrate their consumption for non-alcoholic beverages in the same way as for alcoholic beverages. This might mean that perceptual effects on volume estimation, though present, do not play a central role in determining, and/or do not have a linear influence on, drinking rate for non-alcoholic drinks [20]. As well as volume perception, we explored drink enjoyment. As with a previous study using different sized wine glasses for the consumption of wine [25], we found no evidence that glass shape influenced how much the drink was enjoyed. There was also no association between drink enjoyment and total drinking time. This suggests that shifts in behaviour, including slowing of consumption, might occur without influencing acceptability of the beverage, as found previously for alcohol and warning labels [31].

### Strengths and limitations

There were several strengths to the study. To our knowledge, this study is the first to find an effect of glass shape on drinking behaviour for a soft drink. This extends prior knowledge, suggesting that effects might not be limited to alcoholic drinks. We also used an objective measure of drinking, as well as subjective ratings and perceptual measures (which have previously received relatively more research attention, see [14]). There are also some limitations that should be noted. First, although the portion provided was identical across glass shapes, the glasses could not be matched in fullness, with the inward-sloped glass being less full when holding 330ml than the other two glasses. This confound was largely unavoidable, as manipulating container size (and often shape) inevitably leads to differences in capacity and/or fullness, when keeping other variables (e.g. height) constant. This confound might shed light on why inward-sloped glasses did not appear to slow drinking rate, although further testing is warranted to verify this. Further, the glasses, though matched in height (85-90mm), were not closely matched in weight (varying between 110g-170g). Weight of the drinks container may influence perception of the drink, with some evidence suggesting that heavier vessels *increase* desire for the drink [32], and other evidence suggesting that heavier vessels *decrease* pleasantness ratings [33]. However, given the overall paucity of evidence on the impact of glass weight on perception and/or micro drinking behaviours, it is not clear whether and how the variation in glass weight in our study might have influenced drinking behaviours or perceptions. In addition, we did not measure BMI (which may be relevant here given its link with eating speed [34]). Future studies should ensure BMI is recorded. Finally, it remains to be seen whether and how differences in total drinking time, the outcome variable of interest here, translate into differences in the amount consumed, for example in real world drinking settings when multiple drinks are consumed in a single session.

### Future directions

Given that the effect of glass shape on total drinking time in the current study was substantially smaller than has been previously found using alcohol [20], future studies are required to determine the likely size, as well as parameters, of the effect found. One aim will be to determine the extent to which drink-type (alcoholic vs non-alcoholic) moderates the effect. A second aim will be to explore whether the effect is robust to further changes in the exact glassware used, which will inform as to the exact design elements worth targeting for interventions. A third aim will be to explore the effects of glass shape on drinking behaviour over a longer period, in both laboratory and field settings.

### Conclusion

The current study provides evidence that glass shape influences drinking rate for a soft drink with consumption faster from outward-sloped glasses than from straight-sided glasses. Changes in the micro-structure of drinking–for example, sip size–may be a promising candidate for understanding this effect. In line with a shift of focus towards choice architecture interventions, this study, in conjunction with other experimental and field data, can aid the design of effective interventions to reduce consumption of sugary and alcoholic drinks.

## References

[1] Singh GM, Micha R, Khatibzadeh S, Lim S, Ezzati M, Mozaffarian D. Estimated global, regional, and national disease burdens related to sugar-sweetened beverage consumption in 2010. Circulation. 2015;132(8): 639–66. 10.1161/CIRCULATIONAHA.114.010636 26124185 PMC4550496

[2] Gakidou E, Afshin A, Abajobir AA, Abate KH, Abbafati C, Abbas KM, et al Global, regional, and national comparative risk assessment of 84 behavioural, environmental and occupational, and metabolic risks or clusters of risks, 1990–2016: a systematic analysis for the Global Burden of Disease Study 2016. Lancet. 2017;390(10100): 1345–422. 10.1016/S0140-6736(17)32366-8 28919119 PMC5614451

[3] World Health Organization. Global status report on alcohol and health, 2014; 2014.

[4] Hollands GJ, Marteau TM, Fletcher PC. Non-conscious processes in changing health-related behaviour: a conceptual analysis and framework. Health Psychology Review. 2016;10(4): 381–94. 10.1080/17437199.2015.1138093 26745243 PMC5214381

[5] Marteau TM, Hollands GJ, Fletcher PC. Changing human behavior to prevent disease: the importance of targeting automatic processes. science. 2012;337(6101): 1492–1495. 10.1126/science.1226918 22997327

[6] McGill R, Anwar E, Orton L, Bromley H, Lloyd-Williams F, O’Flaherty M, et al Are interventions to promote healthy eating equally effective for all? Systematic review of socioeconomic inequalities in impact. BMC public health. 2015;15(1): 457. 25934496 10.1186/s12889-015-1781-7 PMC4423493

[7] Department of Health. Healthy lives, healthy people: Our strategy for public health in England, London: Stationery Office; 2010.

[8] Thaler R, Sunstein CR. Nudge: Improving decisions about health, wealth, and happiness. New Haven: Yale University Press; 2008.

[9] Shah M, Schroeder R, Winn W, Adams‐Huet B. A pilot study to investigate the effect of plate size on meal energy intake in normal weight and overweight/obese women. Journal of Human Nutrition and Dietetics. 2011 12;24(6):612–5. 10.1111/j.1365-277X.2011.01210.x 21981018

[10] Rozin P, Scott S, Dingley M, Urbanek JK, Jiang H, Kaltenbach M. Nudge to nobesity I: Minor changes in accessibility decrease food intake. Judgment and Decision Making. 2011 6;6(4):323–32.

[11] Grech A, Allman‐Farinelli M. A systematic literature review of nutrition interventions in vending machines that encourage consumers to make healthier choices. Obesity reviews. 2015 12;16(12):1030–41. 10.1111/obr.12311 26593221

[12] Hollands GJ, Bignardi G, Johnston M, Kelly MP, Ogilvie D, Petticrew M, et al The TIPPME intervention typology for changing environments to change behaviour. Nature Human Behaviour. 2017;1(8): 0140.

[13] Hollands GJ, Shemilt I, Marteau TM, Jebb SA, Kelly MP, Nakamura R, et al Altering micro-environments to change population health behaviour: towards an evidence base for choice architecture interventions. BMC public health. 2013;13(1): 1218. 24359583 10.1186/1471-2458-13-1218 PMC3881502

[14] Spence C, Wan X. Beverage perception and consumption: The influence of the container on the perception of the contents. Food quality and preference. 2015;39: 131–40.

[15] Spence C, Van Doorn G. Does the shape of the drinking receptacle influence taste/flavour perception? A review. Beverages. 2017;3(3): 33.

[16] Van Doorn G, Woods A, Levitan CA, Wan X, Velasco C, Bernal-Torres C, Spence C. Does the shape of a cup influence coffee taste expectations? A cross-cultural, online study. Food Quality and Preference. 2017:56:201–11.

[17] Hollands GJ, Shemilt I, Marteau TM, Jebb SA, Lewis HB, Wei Y, et al Portion, package or tableware size for changing selection and consumption of food, alcohol and tobacco. Cochrane Database of Systematic Reviews. 2015;14(9). 10.1002/14651858.CD011045.pub2 PMC4579823 26368271

[18] Pechey R, Couturier DL, Hollands GJ, Mantzari E, Munafò MR, Marteau TM. Does wine glass size influence sales for on-site consumption? A multiple treatment reversal design. BMC public health. 2016;16(1): 390. 27268112 10.1186/s12889-016-3068-z PMC4896022

[19] Pechey R, Couturier DL, Hollands GJ, Mantzari E, Zupan Z, Marteau TM. Wine glass size and wine sales: a replication study in two bars. BMC research notes. 2017;10(1): 287 10.1186/s13104-017-2610-0 28760155 PMC5537941

[20] Attwood AS, Scott-Samuel NE, Stothart G, Munafò MR. Glass shape influences consumption rate for alcoholic beverages. PloS one. 2012;7(8): e43007 10.1371/journal.pone.0043007 22912776 PMC3422221

[21] Troy DM, Attwood AS, Maynard OM, Scott-Samuel NE, Hickman M, Marteau TM, et al Effect of glass markings on drinking rate in social alcohol drinkers. The European Journal of Public Health. 2016;27(2): 352–356. 10.1093/eurpub/ckw142 PMC5398342 28339526

[22] Lawless HT, Bender S, Oman C, Pelletier C. Gender, age, vessel size, cup vs. straw sipping, and sequence effects on sip volume. Dysphagia. 2003;18(3): 196–202. 14506985 10.1007/s00455-002-0105-0

[23] Bennett JW, Van Lieshout PH, Pelletier CA, Steele CM. Sip-sizing behaviors in natural drinking conditions compared to instructed experimental conditions. Dysphagia. 2009;24(2): 152–158. 10.1007/s00455-008-9183-y 18841414

[24] Zlatevska N, Dubelaar C, Holden SS. Sizing up the effect of portion size on consumption: a meta-analytic review. Journal of Marketing. 2014;78(3): 140–154.

[25] Zupan Z, Pechey R, Couturier DL, Hollands GJ, Marteau TM. Micro-drinking behaviours and consumption of wine in different wine glass sizes: a laboratory study. BMC psychology. 2017;5(1): 17 10.1186/s40359-017-0183-2 28602159 PMC5467050

[26] Cliceri D, Petit E, Garrel C, Monteleone E, Giboreau A. Effect of glass shape on subjective and behavioral consumer responses in a real-life context of drinking consumption. Food Quality and Preference. 2018;64: 187–191.

[27] Maynard Maynard OM, Langfield T, Attwood AS, Allen E, Drew I, Votier A, Munafò MR. No impact of calorie or unit information on ad libitum alcohol consumption. Alcohol and Alcoholism. 2017 9 18;53(1):12–9. 10.1093/alcalc/agx066 PMC5860256 29016721

[28] Bland JM, Altman DG. Transformations, means, and confidence intervals. BMJ: British Medical Journal. 1996;312(7038): 1079 8616417 10.1136/bmj.312.7038.1079 PMC2350916

[29] Olivier J, Johnson WD, Marshall GD. The logarithmic transformation and the geometric mean in reporting experimental IgE results: what are they and when and why to use them?. Annals of Allergy, Asthma & Immunology. 2008;100(4): 333–337 10.1016/S1081-1206(10)60595-9 18450118

[30] Troy D, Attwood AS, Maynard OM, Scott-Samuel NE, Hickman M, Woods A, et al Does glass shape influence the pouring accuracy of liquid volume?; 2017 Preprint. Available from: https://psyarxiv.com/qrufv. Cited 8 Mar 2018. 10.1371/journal.pone.0204562 PMC6198940 30352072

[31] Stafford LD, Salmon J. Alcohol health warnings can influence the speed of consumption. Journal of Public Health. 2017;25(2): 147–154. 10.1007/s10389-016-0770-3 28357194 PMC5350209

[32] Kampfer K, Leischnig A, Ivens BS, Spence C. Touch-flavor transference: Assessing the effect of packaging weight on gustatory evaluations, desire for food and beverages, and willingness to pay. PloS one. 2017 10 11;12(10):e0186121 10.1371/journal.pone.0186121 29020078 PMC5636136

[33] Maggioni E, Risso P, Olivero N, Gallace A. The effect of a container's weight on the perception of mineral water. Journal of sensory studies. 2015 10 1;30(5):395–403.

[34] Ohkuma T, Hirakawa Y, Nakamura U, Kiyohara Y, Kitazono T, Ninomiya T. Association between eating rate and obesity: a systematic review and meta-analysis. International journal of obesity. 2015 11;39(11):1589 10.1038/ijo.2015.96 26100137

